# The Role of Dissociation in the Cycle of Violence

**DOI:** 10.1007/s10896-013-9568-z

**Published:** 2014-02-02

**Authors:** Nicole V. Daisy, Denise A. Hien

**Affiliations:** 1 Women’s Health Project, St. Luke’s/Roosevelt Hospital, New York, NY USA; 2 Derner Institute of Advanced Psychological Studies, Adelphi University, Garden City, NY USA; 3 On Course Psychological Counseling, P.C., 165 N. Village Avenue, Suite 200, Rockville Centre, NY 11570 USA

**Keywords:** Child maltreatment, Dissociation, Substance use, Intimate partner violence, Cycle of violence, Interpersonal trauma, Family violence

## Abstract

The primary aim of this study was to examine the role of dissociation in the relationship between child maltreatment and intimate partner violence among 148 inner city women. It was proposed that dissociation would be a mediator in the relationship between child maltreatment and intimate partner perpetration. Overall, the hypothesis was supported. Findings revealed that women with a history of child maltreatment who experienced high levels of dissociation were more likely to be perpetrators of intimate partner violence than those with low levels of dissociation.

According to the United States Department of Health and Human Services (2010), in 2009 the Administration for Children and Families documented that 3.6 million referrals were investigated by Child Protective Services, with approximately 702,000 substantiated cases of child maltreatment. Clinical and research literature on child maltreatment suggests that experiencing childhood physical abuse, sexual abuse, and witnessing interparental violence may contribute to the propensity to perpetrate intimate partner violence in adulthood (Kwong et al. 2003; Ehrensaft et al. 2003; Heyman et al. 2002). However, there are other risk factors that need to be considered as not everyone who experiences child maltreatment continues the cycle of violence.

There is a growing body of research that has explored the role of substance use as a risk factor associated with the cycle of violence. Research shows that victims of child maltreatment are at risk for using substances later in life (Jasinski et al. 2000; Kendler et al., 2000; Marcenko, Kemp & Larson, 2000). Research also shows that there is an association between substance use and intimate partner violence, specifically perpetration, (Stalans & Ritchie, 2008; Lipsky et al. 2005; Chase et al. 2003; Schafer et al. 2004; Chermack et al. 2001; Stuart et al. 2004; Murphy et al. 2001).

According to the U.S. Department of Justice (2005), between 1998 and 2002, there were 3.8 million offenders of intimate partner violence in both dating and married couples, 41 % of which were under the influence of drugs or alcohol at the time of the incident. Those who use alcohol and drugs are often more susceptible to perpetrating violence (Fals-Stewart, 2003) toward their partners. However, not everyone who uses substances is a perpetrator of intimate partner violence. An underlying risk factor, such as dissociation, may further differentiate those who are unable to break the cycle of violence, since dissociative symptoms are sometimes present among substance users and perpetrators of interpersonal trauma.

Alcohol and certain drugs, such as barbiturates, cannabinoids, benzodiazepines, serotonogeric hallucinogens and general anesthetics, can induce dissociative disorders, particularly derealization, depersonalization, amnesia, and fugue states (Good, 1989). This was supported by a clinical study conducted by Ellason and Ross (1996) in which inpatient adults (*N* = 106) who were admitted due to chemical dependency were assessed for dissociation and psychopathology after they completed the detoxification phase of treatment. The authors found that dissociative disorders were one of the most common forms of comorbidity among former drug and alcohol inpatients with histories of child maltreatment.

Children who are victims of repeated trauma may dissociate to mentally escape (Terr, 1991). Child maltreatment is associated with dissociation (Sandberg & Lynn, 1992; Bagley et al. 1995). Theories on dissociation suggest that difficulty compartmentalizing the traumatic events results in thoughts, emotions, identity, and/or memory not being integrated within the self. Pathological dissociation can be conceptualized as difficulty integrating one’s affect and processing emotional events (Hall, 2003). While peritraumatic dissociation (dissociating during the traumatic event) can help the victim cope with the horrifying situation, pathological dissociation can be detrimental in various ways, including the increased likelihood of perpetrating violence (Simoneti et al. 2000). Finley et al. (2010) examined partner violence of trauma victims and found an increased risk for demonstrating anger in what appears to be a dissociative state, referred to as “dissociative violence.”

Chu (1992) theorized that when victims of trauma are in the intrusion phase of Post Traumatic Stress Disorder (PTSD), in which they are re-experiencing symptoms, they may be hypervigilant to non-threatening events. Those with histories of interpersonal trauma may be at risk for misinterpreting social cues and experiencing distrust, which may result in frequent misunderstandings and conflicts (Luxenberg et al. 2001). Symptoms of dissociation may manifest themselves when affectionate behavior or anger is shown by a loved one, which may conjure up a mixture of negative emotions. Unexpected painful affects associated with traumatic childhood memories, which have been fragmented and cut off from consciousness for several years, may surface. Difficulty with affect integration coupled with the intense rage often felt by victims of interpersonal trauma may result in aggressive behavior toward others.

The association between substance use and violence has been examined more thoroughly than dissociation and violence, probably since substance use is a more overt behavior that may be observed and studied more readily than dissociation. It is important to emphasize that not every substance user becomes violent. Substance users sometimes experience difficulty regulating emotions, a disinhibition of impulses, and impaired judgment, which is also associated with those with high levels of dissociation. Substance use disorders may be comorbid with dissociative disorders; hence, this study aims to examine the role of dissociative symptoms in the cycle of violence among women while controlling for substance use. Dissociation is expected to be the underlying factor that accounts for the association between child maltreatment and perpetration of intimate partner violence. The main purpose of this study is to explore the hypothesis that dissociation may play a mediating role between a history of child maltreatment and subsequent intimate partner violence (i.e., patterns of perpetration), while controlling for substance use.

## Method

The present study is a substudy of a parent grant examining trauma and both retrospectively- and prospectively-determined predictors of victimization and perpetration among a sample of 333 inner-city women with substance abuse and mood disorders. As part of the parent grant, the following four groups of women were recruited: women with cocaine use disorders, women with mood disorders, women with comorbid cocaine use and mood disorders, and a comparison group of women with no history of substance use or depression.

### Sample

This study retrospectively examined 148 women who were involved in an intimate relationship at the time of the interview. Intimate relationship was operationalized as dating or residing with a partner. Of the 148 women, 33.8 % were married, 30.4 % resided with a male partner, .7 % resided with a female partner, 33.8 % dated a male companion, and 1.4 % dated a female companion. Child maltreatment was operationalized as being comprised of participants with a history of childhood physical abuse, sexual abuse, and/or witnessing interparental violence. There was an overlap of the types of victimization that these women experienced, which hampered the ability to differentiate the type of abuse: 4.9 % (*n* = 5) were sexually abused; 40.19 % (*n* = 41) were physically abused; 18.63 % (*n* = 19) experienced both physical and sexual abuse; 18.63 % (*n* = 19) experienced physical abuse and witnessed interparental violence; 16.67 % (*n* = 17) experienced physical and sexual abuse and witnessed interparental violence; and .98 % (*n* = 1) witnessed interparental violence only.

Participants were categorized into two groups: (a) those with substance use disorders and no history of a mood disorder or (b) women with no history of mood disorders or substance use. These categories yielded 56 participants in the substance use group. The criterion that was necessary to place a participant in the substance use group included having a history of frequent and excessive consumption of drugs and alcohol, without having had a history of a mood disorder, as measured by the Structured Clinical Interview for DSM-III-R/DSM-IV.

The inclusion criteria by which participants from the primary study were included in the present study were as follows: the participant was willing to participate in the study, had a permanent address, and was involved in a long-term relationship. Exclusion criteria were severe symptoms of an organic mental disorder, serious physical ailment or chronic disease, or a mood disorder as measured by the Structured Clinical Interview for DSM-III-R/DSM-IV.

### Procedure

Participants were recruited from the general gynecology clinic (OB/GYN) at St. Luke’s Roosevelt Hospital in New York City. As shown in Table 1, the women recruited for this study were primarily African American and Hispanic and were between the ages of 18 and 60. As part of the screening, eligible women were given a brief description of the study and consent was obtained by those who agreed to participate in an assessment conducted by trained research assistants in private offices to ensure confidentiality. The measures were read to participants, in an effort to account for any reading problems. Upon completion of the study, participants were provided with roundtrip transportation, $25 in the form of a grocery voucher for their participation in the study, a referral list of mental health agencies, and a copy of their signed consent for their record.

**Table 1:** Characteristics of the sample

Demographic variable	Percentage or mean (standard deviation)
Age	32.8 (7.9)
Ethnicity:	
Caucasian	2.7 %
African American	45.9 %
Latino	50.0 %
Native American	1.4 %
Religion^a^:	
Catholic	50.7 %
Protestant	31.8 %
Other	10.1 %
None	6.1 %
Socioeconomic status:	
Monthly income	$846.0 (713.5)
Education:	
Attended grade school	4.7 %
Completed 8th grade	4.1 %
Partial high school	32.4 %
Completed high school	20.3 %
Partial college	31.1 %
Completed college	6.8 %
Completed graduate degree	.7 %
Employment status^a^	
Unemployed	39.2 %
Part-time	19.6 %
Full-time	16.9 %
Disability/retired	2.0 %
Student	4.1 %
Homemaker	16.9 %

*N* = *148*

^a^2 participants did not disclose their religion or employment status

### Measures

#### Demographic and Treatment History Form (Hien & Zimberg, 1991)

This is a structured 62-item interview containing questions regarding demographic and life history information for each participant. Some such information includes family history and an extensive history of psychiatric problems, history of psychiatric hospitalizations, and/or psychiatric treatment.

#### Structured Clinical Interview for DSM-III-R/DSM-IV (SCID) (Spitzer et al. 1992; Revised by Nunes et al., 1996)

The SCID is a structured clinical interview designed to assess lifetime and current major Axis I DSM-IV diagnoses using a decision tree approach. The following modules were utilized for this study: Alcohol and Psychoactive Substance Use Disorders and Mood Disorder.

#### Childhood Trauma Questionnaire (CTQ)-Physical Abuse subscale (Bernstein et al., 1994)

The CTQ is a 70-item questionnaire that assesses the following five factors of abusive experiences during childhood: physical abuse, emotional abuse, physical and emotional neglect, and sexual abuse. The Physical Abuse 23-item Likert subscale was utilized to assess physical and emotional abuse. The items are rated on a 5-point Likert scale according to the frequency of the experiences, ranging from 1 (*never true*) to 5 (*very often true*). This scale has demonstrated strong reliability and validity among patients in treatment for substance abuse (Bernstein et al, 1994).

#### Childhood Sexual Abuse Interview (Wyatt, 1985, modified by Miller, 1990)

This measure asks the participants to recall specific sexual experiences that occurred prior to the age of 18. Perpetrators are defined as someone at least 5 years older than the victim, any relative regardless of age, or someone who initiated these acts against the victim’s will. The following three subscales are created based on the participant’s responses: exposure, touching (manual or oral sexual touching), and penetration (intercourse or digital penetration).

#### Conflict Tactics Scale - Partner Version (CTS-Partner) (Straus, 1979; Revised, Miller, 1990)

The CTS is a self-report measure consisting of 14 items that assess the occurrence of partner violence experienced or perpetrated in the 6 months prior to the interview. Based on responses to the CTS, indices of overall partner violence can be computed and a moderate to severe partner violence subscale rating can be calculated using scoring conventions developed by Straus and Gelles (1986). These indices were modified by Miller (1990) and additional questions for the moderate to severe partner violence subscale were added regarding the use of alcohol or drugs during violent acts, as well as consequences of the violence and family attitudes toward violence. The scale has high construct validity and reliability (Straus & Gelles, 1986).

#### Dissociative Experiences Scale (DES) (Bernstein & Putnam, 1986; Carlson & Putnam, 1993)

The DES is a 28-item self-report questionnaire that assesses problems with memory, attention, identity, and perception. Participants are required to write the amount of time (in percentages that are scored to the nearest 10 %) that they experienced each symptom. The total score is the mean score on the 28 items. This measure yields three of the following subscales: absorption-imaginative involvement (i.e., losing awareness of external events when engrossed in an activity), activities of dissociated states (e.g., finding oneself in an unfamiliar place without awareness of how one arrived there), and depersonalization-derealization (i.e., when one feels that he/she or others are unreal). The DES has demonstrated good reliability and validity among inpatient and outpatient samples (Bernstein & Putnam, 1986).

## Results

Among the 148 women who participated in this study, 102 experienced child maltreatment, which was characterized as women who experienced sexual or physical abuse in childhood or witnessed interparental violence before the age of 18. Descriptive statistics for the Childhood Sexual Abuse Inventory and the Childhood Physical Abuse Scale are presented in Table 2.

**Table 2:** Nature and frequency of the different kinds of abuse experienced

Total reporting physical, sexual or witnessing interparental violence in childhood	*n*	%
	102	68.92
Witnessing interparental violence		
Total	37	25.00
Childhood physical abuse		
Total	96	64.09
Type of physical abuse^a^:		
Corporal punishment	40	41.66
Pushed or shoved	18	18.75
Bruised	16	16.66
Requiring medical attention	4	4.17
Noticed by a mandated reporter or neighbor	9	9.38
Needed to fight or flee for protection	23	23.96
Childhood sexual abuse		
Total	41	28.00
Type of sexual abuse^a^:		
Molestation	34	82.92
Fondling	28	68.29
Exposure to masturbation or showing of genitals	29	70.73
Oral or anal penetration	16	39.02
Pornography	2	4.88
Intercourse	16	39.02
Relationship of perpetrator		
Intrafamiliar:		
Parent or step/foster parent	8	19.51
Sibling	2	4.88
Other relative	11	26.83
Extrafamiliar:		
Family friend or Trusted adult	11	26.83
Friend/peer	4	9.76
Stranger or other	5	12.02
Frequency of the Abuse		
1	14	34.15
2-10	12	29.27
Chronic	15	36.59

^a^Some participants experienced more than one type of abuse

There are two ways of examining dissociation: (a) to characterize each woman in terms of the actual number of symptoms and (b) to dichotomize the women into two groups, high dissociators and low dissociators. When examined as a continuous variable, the mean dissociation was 7.89 with a standard deviation of 9.73. When examined as a dichotomous variable, the dissociation scores were divided into high and low categories using a mean score of 17. This score was chosen because scores between 15 and 20 have been found to be most sensitive for screening dissociative disorders (Steinberg et al. 1991). There were 122 women who had scores of 17 or below and 25 women who had scores greater than 17. Among the 122 women in the low dissociation group, the mean dissociation was only 4.27 (*SD* = 4.22), whereas the dissociation among the 25 women in the high dissociation group was over five times greater (*M* = 25.55, *SD* = 9.74).

Of the 61 women who reported that they were currently involved in an intimate relationship in which violence exists, 52.46 % (*n* = 32) reported that both she and her partner were violent toward each other. Over 30 % (*n* = 45) of women in this sample perpetrated violence toward their current partner (i.e., experienced respondent violence), and 33.33 % (*n* = 16) of these women were not victimized by their partners. Table 3 summarizes the types of incidents that occurred.

**Table 3:** Type of Partner violence among those in the partner violence subgroup

Measure	Partner violence percentage (*n*)	Respondent violence percentage (*n*)
Type of incident^a^:		
Slapped	51.1 (23)	52.1 (25)
Pushed, grabbed or shoved	64.4 (29)	62.5 (30)
Kicked, bitten or punched	37.8 (17)	35.4 (17)
Beaten up	20.0 (9)	8.3 (4)
Raped	11.1 (5)	2.1 (1)
Choked	15.6 (7)	4.2 (2)
Burned	4.4 (2)	0.0
Used a knife or gun	4.4 (2)	4.2 (2)

*N* = *61*

^a^Some participants experienced more than one type of intimate partner violence

### The Cycle of Violence

Participants were asked about their interactions with their partners within the past 6 months to determine their recent experiences; however, they may have experienced intimate partner violence in the past. Of the 102 women who experienced child maltreatment, 50 did not experience any intimate partner violence and 52 were either victimized by their partner or perpetrated violence against their partner. Of these 52 women, 25 % (*n* = 13) perpetrated violence against their partner, and 50 % (*n* = 26) were both victims and perpetrators. Bivariate analyses were conducted to examine the relationships between child maltreatment, substance use, dissociation, and intimate partner violence.

### Associations between Child Maltreatment, Substance Use, and Dissociation

Survivors of child maltreatment were more likely to use substances^1^ than women without a history of child maltreatment. A Chi-square test examined the relationship between child maltreatment and substance use and the results show that the difference between women with and without a history of child maltreatment who used substances was significant, χ^2^ (1, *N* = 148) = 7.35, *p* = .007.

Victims of child maltreatment were more likely to dissociate than women without a history of such trauma. A Mann-Whitney test compared the dissociation, which was characterized as a continuous variable, of women with a history of child maltreatment (*n* = 46) to those without a history of child maltreatment (*n* = 102). The difference between the mean ranks of dissociation among women without a history of child maltreatment (*M* = 55.68) and women with a history of child maltreatment (*M* = 82.34) were significantly different, z = -3.52, *p* < .001.

### Associations Among Substance Use, Dissociation, and Intimate Partner Violence

Women who used substances were more likely to perpetrate violence toward their partner than non-substance users, χ2 (1, *N* = 148) = 12.69, *p* = <. 001. A chi-square test was also applied to the relationship between dissociation (dichotomous variable) and respondent violence and found to be statistically significant, χ2 (1, *N* = 148) = 25.74, *p* = <. 001.

### The Role of Dissociation in the Cycle of Violence

Multiple regression analyses were performed to test the hypothesis that dissociation mediates the relationship between child maltreatment and intimate partner violence. Prior to conducting the mediation analyses, correlational analyses were used to examine potential demographic covariates. Pearson’s correlation showed a significant association between dissociation and not having a college education, *r*(147) = -0.23, *p* < .01); not being married, *r*(147) = -0.21, *p* < .05); and being unemployed. *r*(147) = 0.19, *p* < .05. Pearson’s correlation showed a significant association between respondent violence and a young age, *r*(61) = -.17, *p* < .05; not having a college education, *r*(61) = -.22, *p* < .01; and having children, *r*(61) = -.35, *p* < .001. Next, in an effort to more closely examine the relationship between the main measures and these demographic variables, regressions were conducted. A linear regression analysis revealed that having no college education was a significant predictor of dissociation (*B* = -.23, *p* < .01). Having a history of substance use, no college education, and being a parent were significant predictors of respondent violence (*B* = .23, *p* < .01; *B* = -.19, *p* < .05; *B* = -.38, *p* < .001 respectively). The aforementioned covariates that were significant predictors of the outcome variables were used in the subsequent mediation analyses.

Child maltreatment was a predictor variable, which was a continuous measure comprised of the sum of exposure to childhood sexual abuse, physical abuse, and having witnessed interparental violence. Dissociation was characterized as a continuous variable, which included the sum of dissociative symptoms. Respondent violence was the criterion variable, which was also characterized as a continuous variable that was comprised of the sum of instances of perpetration of violence toward their partner. Substance use was controlled for within the following regression analyses since it is a confounding variable with dissociation.

A stepwise multiple regression was performed to test the hypothesis that dissociation mediates the relationship between child maltreatment and intimate partner violence. According to Baron and Kenny (1986), there are a series of regression analyses that need to be completed in order to confirm a mediational hypothesis. Initially, the mediator will be regressed onto the predictor variable. Next the criterion variable will be regressed onto the predictor variable. Finally, the criterion variable will be regressed onto both the predictor and mediator variables.

A linear regression was conducted to examine whether or not child maltreatment is a significant predictor of dissociation. Substance use and education were covariates, since they were significant predictors of dissociation. Findings revealed that child maltreatment was a trend (*B* = 0.12, *p* = .14). Table 4 summarizes these findings.

**Table 4:** Linear regression analysis predicting dissociation from child maltreatment

Step and predictor variable	R square	R sq change	*F*	*p* value	Standardized beta	*p* value
Regression 1 (Covariates)	.16	.16	13.26	<.001***		
Substance use					.34	<.001***
Education					-.14	.088**
Regression 2	.17	.01	9.64	<.001***		
Substance use					.31	<.001***
Education					-.13	.098**
Child maltreatment					.12	.141^+^

*N* = *147* ^+^ *p* < = .1 **p < .01 ***p < .001

Next, linear regressions were conducted to examine the relationship between child maltreatment and respondent violence. Substance use, education and number of children were covariates since they were predictive of respondent violence. As shown in Table 5, findings revealed that child maltreatment was a trend (*B* = 0.11, *p* = .15).

**Table 5:** Results of linear regressions predicting respondent violence from child maltreatment

Covariate	*F*	*p* value	R square	R square change	B	Standardized beta	*p* value
Substance use	13.43	<.001	.23	.14	.84	.23	.005**
Education					-.72	.30	.12*
Number of children					-2.25	-.38	<.001***
Substance use	10.67	<.001	.24	.24	.77	.21	.10*
Education					-.65	-.17	.033*
Number of children					-2.21	-.37	<.001***
Child Maltreatment					.14	.11	.15^+^

*N* = *137* ^+^ *p* < = .1 *p < .05 **p < .01 ***p < .001

Last, linear regressions were used to examine the role of dissociation in the relationship between child maltreatment and respondent violence. As shown in Table 6, dissociation accounted for 27 % of the variability in respondent violence. Findings showed that dissociation was a statistically significant predictor of respondent violence and that the significance of child maltreatment decreased, as evidenced by the smaller standardized beta. The decrease in the level of significance for the child maltreatment variable suggests that dissociation is mediating the relationship between child maltreatment and intimate partner violence.

**Table 6:** Results of linear regressions predicting respondent violence from child maltreatment and dissociation

Covariate	*F*	*p* value	R square	R square change	B	Standardized beta	*p* value
Substance use	13.43	<.001	.23	.14	.84	.23	.005**
Education					-.72	.30	.12*
Number of children					-2.25	-.38	<.001***
Substance use	10.67	<.001	.24	.24	.77	.21	.10*
Education					-.65	-.17	0.03*
Number of children					-2.21	-.37	<.001***
Child Maltreatment					.14	.11	.15^+^
Substance use	9.81	<.001	.27	.27	.57	.15	.06
Education					-.57	-0.15	0.06
Number of children					-2.22	-0.37	<.001***
Child maltreatment					9.48	0.08	0.32
Dissociation					3.72	0.19	0.03*

Since interpersonal trauma in adulthood can lead to manifestation of dissociative symptoms, exposure to community violence was controlled in an effort to exclusively examine the role of child maltreatment in dissociation and intimate partner violence. Controlling for community violence in adulthood did not change the role of dissociation in the cycle of violence

*N* = *137* ^+^ *p* < = .1 *p < .05 **p < .01 ***p < .001

## Discussion

This study examined the role of dissociation in the relationship between child maltreatment and intimate partner violence while controlling for substance use. Substance use was associated with the dependent, mediator, and independent variables; however, dissociative symptoms were also present, which warranted further examination. Overall, the results of this study indicated that dissociation is a mediator between child maltreatment and respondent violence.

This study contributes to the volume of extant research by exploring how victims of abuse, who may have turned to substance use, may need help reducing symptoms of dissociation, which in turn may result in reduced likelihood of perpetrating intimate partner violence. The findings of this study show that victims of child maltreatment may manifest symptoms of dissociation and have patterns of behavior that can affect one’s ability to utilize non-violent conflict resolution skills in their intimate relationship. Thus, dissociation in conjunction with substance use can contribute to our understanding of the cycle of intrafamilial violence.

Results of this study support previous findings of an association between child maltreatment and intimate partner violence (Kwong et al., 2003; Ehrensaft et al., 2003; Heyman et al. 2002). Findings also revealed that if a woman had a history of child maltreatment, she was more likely to use substances. This finding suggests that trauma victims may disconnect from painful affect associated with the trauma by seeking an escape with drugs and alcohol, which supports research regarding the relationship between trauma and substance use (Dayton, 2000). The finding that substance use is associated with intimate partner violence contributes to the previous research that has linked substance use with violent behavior (McMurran, 1999; Schafer et al., 2004). Although substance use is associated with interpersonal trauma among women in this study, it is important to note that not every substance user is a victim or perpetrator of intimate partner violence. The results of the study showed that an underlying factor, such as dissociation, may contribute to the cycle of violence.

As previously discussed, several studies have found that substance users may have a history of trauma, but understanding what makes some trauma survivors resort to substance use and others more resilient may lie in the level of dissociative symptoms that they manifest. Among the women in this study, substance users manifested more dissociative symptoms than non-substance users. The mean dissociation for the substance using group was 12.6 (*SD* = 12.5) whereas the non-substance using group had a mean of only 5.1 (*SD* = 6.2). The mean dissociation for the non-substance using group was similar to non-substance using groups in other studies, such as Bernstein and Putnam (1986), in which the mean was 4.38; however, the mean dissociation for the substance use group was 12.6 (*SD* = 12.5) was lower than the mean in other studies, such as Ross et al. (1992) and Dunn et al. (1993), in which the means were 14.7 and 15.40, respectively. The high amount of dissociative symptoms manifested by substance users within this study demonstrates the need for clinicians to assess a client’s level of dissociation once substance use is reported.

Victims of child maltreatment were more susceptible to experiencing dissociative symptoms. This finding is consistent with researchers (Luxenburg et al. 2001; Terr, 1991) who have given recognition to the elevated dissociative states experienced by trauma victims. Findings from this study also support previous studies that found an association between dissociation and the perpetration of violence in intimate relationships (Simonetti et al. 2000; Finley et al., 2010).

There are characteristics of the design of this study that warrant consideration. Due to the correlational design of the study, it is impossible to determine causality. The results may have been confounded by an overlap of factors; for example, some of the women in this study were both victims and perpetrators, which make it difficult to assess whether childhood traumas or current traumas are the reason for the participants’ dissociation. Another limitation is the way that mediation was examined. A more recent approach to examining mediation, such as bias corrective bootstrapping, would have strengthened the results of this study.

Another limitation worth discussing is that the histories are based on self-report. When self-report is the only measure of a participant’s history, there is a chance that the participant may have under-reported the amount and type of substances used, may find it difficult to disclose painful memories, or may have an altered perception of the length of exposure to the trauma.

A final limitation was that the sample was comprised of high-risk urban women with a low socioeconomic status, which makes it difficult to generalize. One of the challenges of this study was examining specific types of trauma among a population that was exposed to various types of trauma in the environment. Examining multiple interpersonal traumas was difficult because a single episode interpersonal trauma could be accompanied by exposure to several situational traumas.

Examining different types of perpetration, such as perpetrating child maltreatment toward their own children, was beyond the scope of this study but warrants further examination. Findings of the high rates of trauma exposure among this population of inner city women warrant a need for therapists to explore trauma history under the rubric of drug treatment, especially since minimizing the trauma or having fragmented memories of the trauma is likely among high dissociators. Finally, while this study sought to examine the factors that led to the cycle of violence, examining the factors associated with those that broke the cycle of violence would be beneficial. An extensive assessment of factors that may have assisted women to be resilient was beyond the scope of this study but warrants further examination.

## Footnotes

1 The substance-using women in this study had lifetime histories of substance use disorders but were not necessarily currently using substances. However, their history of substance use suggests that they currently experience difficulty with emotion regulation, which are posited to exist regardless of whether or not they are currently using substances.
